# Editorial: Long COVID: nutrition and lifestyle changes

**DOI:** 10.3389/fnut.2024.1375449

**Published:** 2024-02-21

**Authors:** Germano Guerra, Angela Lucariello, Klara Komici

**Affiliations:** ^1^Department of Medicine and Health Sciences, University of Molise, Campobasso, Italy; ^2^Department of Sport Sciences and Wellness, University of Naples “Parthenope”, Naples, Italy

**Keywords:** Long COVID, COVID-19, vitamin D, antioxidants, lifestyle interventions

Long COVID is a clinical condition that occurs in individuals with an incomplete recovery from SARS-CoV-2 infection. Long COVID, also known as “post-acute sequelae of COVID-19 (PASC)”, affects multiple systems including the neurological, cardio-respiratory, gastrointestinal, and musculoskeletal systems (1). Myalgia, fatigue, shortness of breath, chest pain, cognitive impairment, and headache are the most frequent symptoms. The persistence of symptoms is at least 12 weeks following acute COVID disease and Long COVID is a diagnosis of exclusion (2, 3). The underlying mechanisms remain largely unknown; however, oxidative stress, chronic inflammation, and gut dysbiosis can contribute to the pathogenesis of Long COVID (4, 5). Exercise intolerance, dependency, mood disorders, and a reduction of quality of life may be the consequences of Long COVID. The present research is insufficient to provide interventions which ameliorate the conditions and the outcome of patients with Long COVID (1, 3). However, flavonoids ameliorated olfactory dysfunction and memory clouding in patients with Long COVID (6). A low vitamin D level was associated with Long COVID syndrome (7). L-Arginine and vitamin C supplementation attenuated typical symptoms of Long COVID (8). A prospective study showed that gut microbiota composition was associated with the occurrence of PACS (9). Vitamins, nutraceuticals, and probiotics participate in the response of immune system, modify oxidative stress and inflammation, and may alleviate Long COVID symptoms (10, 11). Furthermore, a recent meta-analysis reported that rehabilitation was associated with a significant improvement in exercise capacity and quality of life compared to standard of care (12). Based on these considerations, in this Research Topic, we aimed to collect articles that describe the role of nutritional, dietary, physical activity, and all lifestyle interventions on Long COVID.

Matías-Pérez et al. summarized the role of quercetin on oxidative stress. Quercetin belongs to the class of flavonoids and neutralizes free radicals, inhibits NADPH oxidase, and enhances the production of endogenous antioxidants. Furthermore, it inhibits the binding of viral capsid proteins and controls the production of proteases and polymerase which is important for the control of viral load. The authors suggested that consumption of quercetin may decrease the SARS-CoV-2 viral load, the release of pro-inflammatory cytokines, and the production of mucus from the respiratory system. It was underlined that a maximum consumption of 1,500 g daily would not show harmful effects.

Aghajani et al. explored the association of a dietary antioxidant quality score (DAQS) and the severity of COVID-19 disease in a case-control study. To 104 intensive care unit patients and 191 patients without severe complications, a 147-item semi-quantitative food frequency questionnaire was administrated. The results indicated a significant association between vitamin D intake and decreased risk of COVID-19 severity: OR = 0.91; 95% CI: 0.89–0.94, *p* < 0.001. This association was significant considering potential confounders such as age, body mass index (BMI), and other dietary components. Vitamin D may downregulate the release of Interleukin 6 and TNF-alpha and may increase the T regulatory lymphocyte levels (13, 14). Of interest, a significant association between vitamin C intake and the risk of COVID-19 infection severity was observed only in female patients: OR = 1.00; 95% CI: 1.00–1.00, *p* = 0.028. It should be mentioned that vitamin C may control the cytokine storm in the alveolar region and may deplete neutrophil extracellular trap formation which are relevant for COVID-19 (11).

Grundler et al. described a case-series of 14 patients with Long COVID who underwent a medically supervised long-term fasting according to the Buchinger Wilhelmi protocol (15). In this study, the patients were admitted for 10–21 days and underwent a fasting therapy for 6–16 days, with a subsequent food reintroduction period of 3–5 days. Other interventions as physiotherapy, psychotherapy, ozone therapy, and acupuncture were introduced as well. No adverse events were reported. Reduction of body weight, systolic blood pressure, and amelioration of glycemic and lipid profile was observed. Data regarding inflammatory biomarkers such as high sensitivity C-reactive protein and erythrocyte sedimentation rate were inconsistent. Self-care, mobility, usual activities, pain, anxiety and mood improved in most of the patients. Improvements in common symptoms like headache, fatigue, weakness, shortness of breath, muscle pain, joint pain, and sleep difficulties were registered. Long fasting and caloric restriction have been shown to enhance oxidative stress defense, anti-inflammatory response, and improve the metabolic profile and cardiovascular risk (16, 17). While fasting improved survival in experimental models of bacterial inflammation (18), the safety of fasting duration raises concerns.

The measurement of body composition is important for the evaluation of nutritional status. das Virgens et al. provided a scoping review to describe how body composition was assessed in patients with COVID-19 and what the impact of body composition was on disease severity and in hospital outcome. Computer tomography, bioelectrical impedance analysis (BIA), and ultrasound (CT) were the main methods used in 55 included studies. Fat mass, muscle mass, and phase angle were significant indicators of the severity of disease, length of hospitalization, and mortality. We have demonstrated that body composition is an independent predictor of respiratory function (19, 20) and increased fat mass results in a reduction of cardio-respiratory capacity (20), which in turn is a well-established parameter of overall survival. Little evidence is present regarding the modification of muscle mass during COVID-19 disease.

The articles collected in this Research Topic suggest that a higher intake of dietary antioxidants and vitamin D is associated with lower risk for COVID-19 severity. Considering the antioxidant properties of quercetin, supplementation with quercetin should be explored for alleviation of Long COVID symptoms. Further research is necessary to establish if gender-specific recommendations should be applied for supplementation with dietary antioxidants. Body composition may predict the severity and outcomes of COVID. Modification of body composition related to Long COVID should be explored. Long fasting improves the overall perception of health in individuals with Long COVID but the results regarding the inflammatory profile are still inconsistent. Future clinical trials should focus on the benefits and safety of long fasting in patients suffering Long COVID syndrome.

## Author contributions

GG: Writing—original draft. AL: Writing—original draft. KK: Writing—original draft.
